# Resistance to drugs and cell death in cancer stem cells (CSCs)

**DOI:** 10.15761/jts.1000341

**Published:** 2019-06-24

**Authors:** Ahmad R Safa

**Affiliations:** Department of Pharmacology and Toxicology, Indiana University School of Medicine, Indianapolis, USA

**Keywords:** cancer stem cells (CSCs), apoptosis, drug resistance, death receptor pathways, anti-apoptotic proteins, Bcl-2 family, c-FLIP

## Abstract

Human cancers emerge from cancer stem cells (CSCs), which are resistant to cancer chemotherapeutic agents, radiation, and cell death. Moreover, autophagy provides the cytoprotective effect which contributes to drug resistance in these cells. Furthermore, much evidence shows that CSCs cause tumor initiation, progression, metastasis, and cancer recurrence. Various signaling pathways including the phosphatidylinositol 3-kinase (PI3K)/Akt/mammalian target of rapamycin (mTOR), maternal embryonic leucine zipper kinase (MELK), NOTCH1, and Wnt/β-catenin as well as the CSC markers maintain CSC properties. Several mechanisms including overexpression of ABC multidrug resistance transporters, a deficiency in mitochondrial-mediated apoptosis, upregulation of c-FLIP, overexpression of anti-apoptotic Bcl-2 family members and inhibitors of apoptosis proteins (IAPs), and PI3K/AKT signaling contribute to enhancing resistance to chemotherapeutic drugs and cell death induction in CSCs in various cancers. Studying such pathways may help provide detailed understanding of CSC mechanisms of resistance to chemotherapeutic agents and apoptosis and may lead to the development of effective therapeutics to eradicate CSCs.

## Introduction

**Figure 1.**
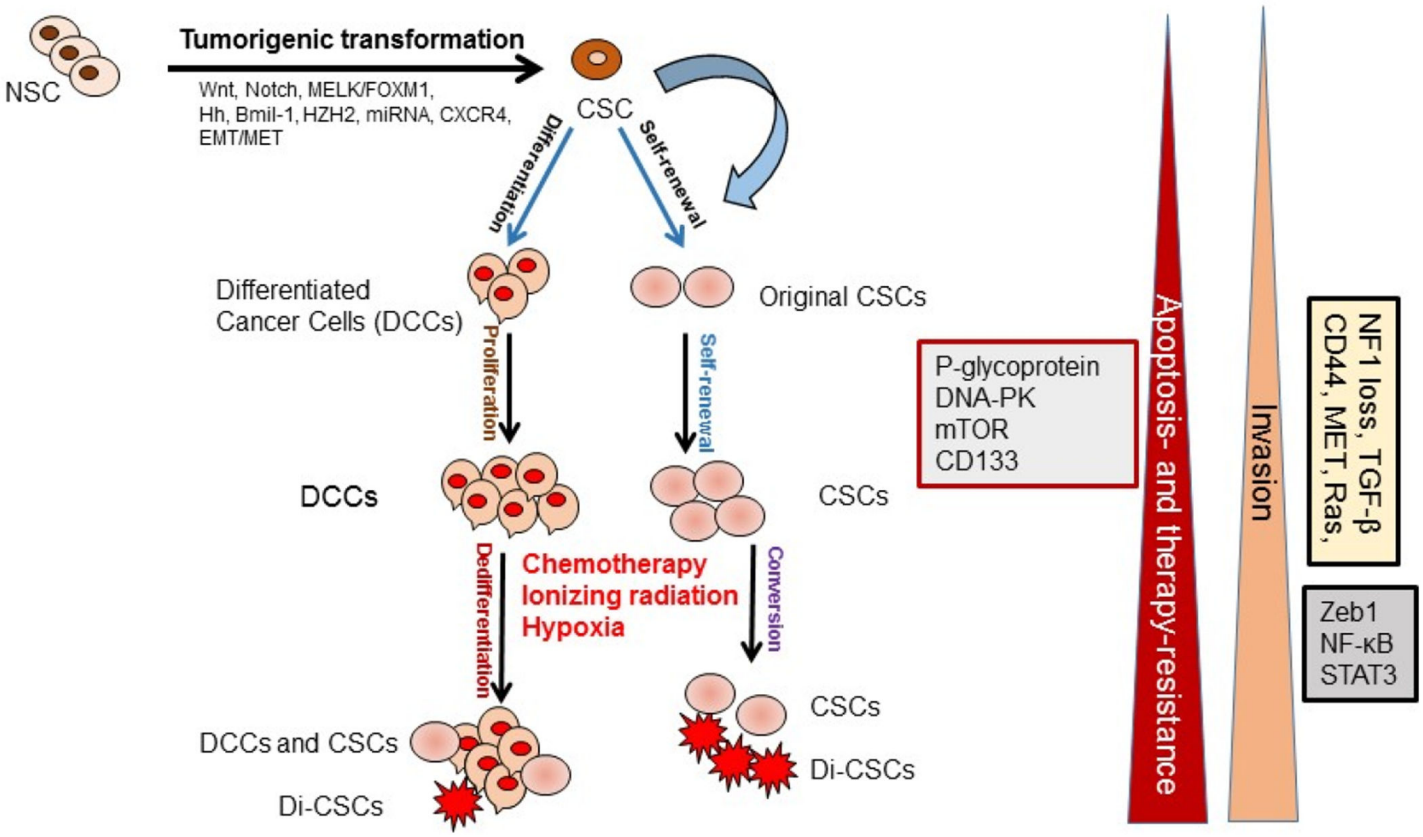
Cancer stem cells (CSCs) role in tumor development and progression. CSCs are generated from the normal stem cells (NSCs) through tumorigenic transformation of several potential pathways including Hh: hedgehog, epithelial-to-mesenchymal transition (EMT), and the reverse process mesenchymal-to-epithelial transition (MET). CSCs and drug-induced CSCs (Di-CSCs) are enriched following conventional chemotherapy treatment

Various cancer types contain a highly heterogeneous cell population containing distinct subpopulations of cells with genetic, epigenetic, and morphologic variations. The heterogeneity and progression of malignancies are explained by two models: (a) the CSC or cancer initiating cell (ClC) model [1,2], and (b) the stochastic model or clonal evolutionary model [3]. The CSC model or hierarchical model describes that tumors arise from a small percentage of CSCs as a result of normal stem cell (NSC) mutations and strongly suggests that CSCs cause an orderly system generating the entire tumor cell population [1,2] (Figure 1). In the stochastic model, normal cells acquire mutations during their survival before they develop into CSCs and initiate tumorigenesis [3]. Moreover, CSC-like cells can be generated by epigenetic plasticity due to drug-induced dedifferentiation or conversion of non-CSCs to CSCs, which make cancer therapy more complex [4] (Figure 1). However, in the clonal evolutionary model, each cell within the tumor possesses the potential to produce malignant tumors expressing various degrees of chemoresistant subpopulations (Figure 1).

**Figure 2.**
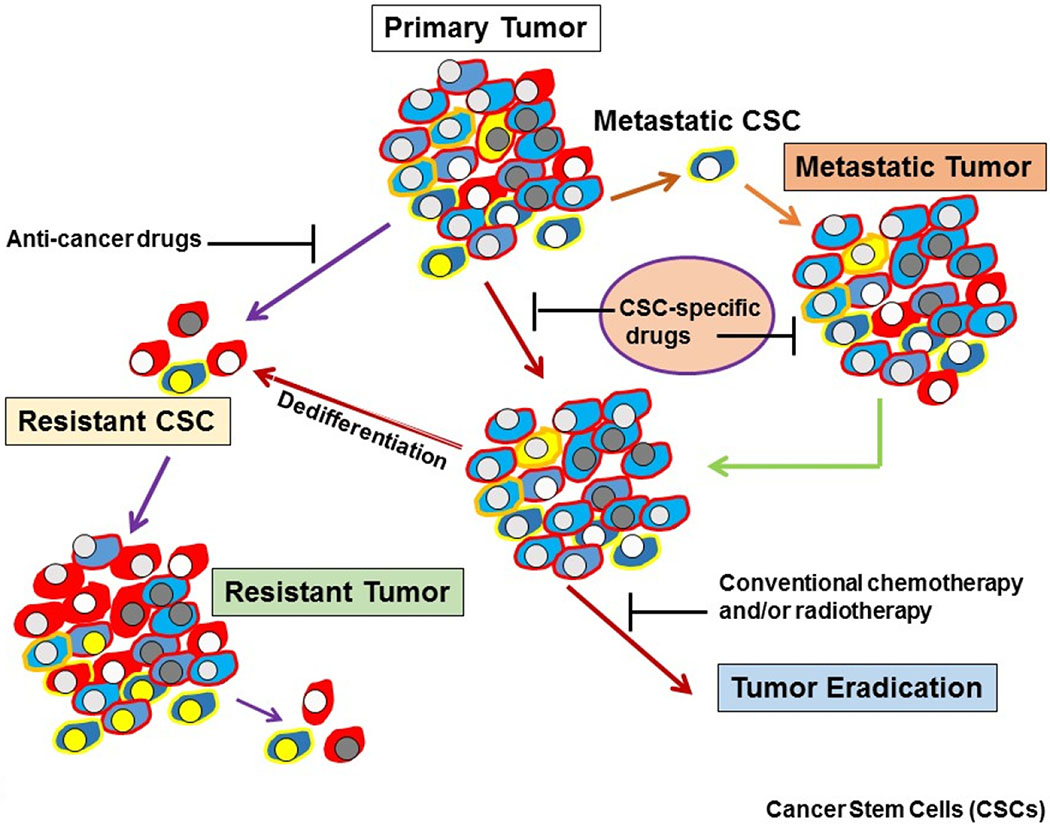
Heterogeneity of CSCs in tumors. Development of drug resistant, metastatic tumors, and a potential strategy for eradicating tumors using CSC-specific drugs

Tumor recurrence is the major cause of death in patients with incurable malignancies and is attributed to treatment-resistant CSCs within the primary tumor (Figure 2). CSCs are a rare cell population within a tumor, which express differing molecular markers in various types of cancers [1,2,4]. Delineating the molecular characterization of CSC populations will allow us to identify the targeted agents that cause their cell death, thus increasing the development of more effective treatment regimens for cancer. In this model, tumors contain a very small population of tumor initiating cells termed CSCs or stem-like cells, which are responsible for the initiation, progression, metastasis, drug resistance, and recurrence of cancer [1,2,4]. These therapy resistant, quiescent and pluripotent cells reside in CSCs niches, which provide specific microenvironments that protect CSCs against cell death, chemotherapy, and radiotherapy [5,6]. Therefore, there is a hierarchy of cells within tumors, which is initiated from a CSC population. Tumors exhibit stemness (self-renewal and multilineage differentiation) because CSCs have the capacity to recapitulate xenografts similar to the original tumor [5,6]. The self-renewal and differentiation capacities lead to the production of various cancer cell types in tumors and thereby create tumor heterogeneity [7] with various degrees of resistance to different therapeutics.

Drug resistance has been a major impediment for successfully treating tumors, not only with conventional chemotherapeutics, but with targeted therapies as well [8–11]. A major contributor to drug resistance is cellular heterogeneity within a tumor. Another main limitation in cancer therapy has proven to be a lack of refractoriness to apoptosis due to intrinsic resistance to cell death (e.g., pancreatic cancer, colon cancer, glioblastoma, and prostate cancer are typically refractory to cancer chemotherapy) or acquired resistance (e.g., after breast cancer chemotherapy). Much evidence has proven that within solid tumors, there are distinct populations of cancer cells contributing to the complexity of cancer treatment. Major contributors to intratumoral heterogeneity are cancer stem cells (CSCs), cellular genotype, genomic instability, epigenetic variation, cell plasticity, stochastic processes, and the microenvironment including distinct subpopulations of cancer associated fibroblasts (CAFs) and cancer-associated macrophages (CAMs) [12] causing various effects on cancer cells. Therefore, drug resistance in tumors is very complex and modulation or circumvention of drug resistance requires specific inhibitors for targeting relevant cellular targets.

Chemoresistance emerges due to several factors including environmental factors, pharmacodynamics, tumor heterogeneity in each patient as well as genetic and epigenetic alterations in malignant cells [13–17]. Many mechanisms participate in triggering resistance to chemotherapeutic drugs in cancer cells and characterizing these mechanisms will provide critical information for the design and development of more effective approaches to circumvent drug resistance in malignant cells and tumors. Deregulation of apoptotic and cell death signaling pathways and upregulation of survival mechanisms in cancer cells, particularly in CSCs, confer resistance to various drugs in a wide variety of cancers [18–22]. Additionally, epigenetic alterations can contribute to drug resistance and aggressive tumor behavior. Therefore, designing approaches to increase cell death in CSCs may lead to the development of useful therapeutics to eliminate these cells. Moreover, epigenetic plasticity is specifically critical in the interconversion of differentiated cells into CSCs and vice versa [4], which adds to the complexity of successfully treating cancers. Therefore, the major requirement for effective and successful cancer therapeutics is to eliminate CSCs, differentiated cancer cells, and the heterogeneous subpopulations of cancer cells in the entire tumor.

## Drug resistance in CSCs

Strong evidence shows that CSCs are highly resistant to conventional chemotherapies [11,23–32]. Various drug resistance mechanisms have been reported in CSCs including increased anti-apoptotic proteins such as Bcl-2 Bcl-X, and c-FLIP [11,26], high expression of ATP-binding cassette (ABC) transporter proteins and detoxifying enzymes [26–28], cell cycle quiescence [29,30], increased DNA repair ability [26,27], elevated aldehyde dehydrogenase (ALDH) activity [31], activation of key prosurvival signaling molecules such as NOTCH, Wnt/β-catenin, and NF-κB [32–34], increased activities of the phosphatidylinositol 3-kinase (PI3K)/Akt/mammalian target of rapamycin (mTOR), and maternal embryonic leucine zipper kinase (MELK) [11,35].

Recent findings show that CSCs are quiescent in the resting stage of the cell cycle and resistant to chemotherapy since most of these drugs target proliferating cells [36,37]. Upregulation of DNA repair proteins in CSCs correlates with rapid DNA repair, which also participates in drug and radiation resistance. [27,38–40]. Moreover, the cancer microenvironment (niche) protects CSCs from cancer therapy [27,41] and CSCs contribute to the niche in a feedback loop and mutual manner [32,41]. The extracellular matrix (ECM) facilitates and maintains cancer stem cells and drug resistance [42]. In addition to delineating molecular and biochemical mechanisms of drug resistance, understanding and defining the crosstalk between CSCs and their niche is critical for overcoming resistance to anticancer drugs and cell death.

**Figure 3.**
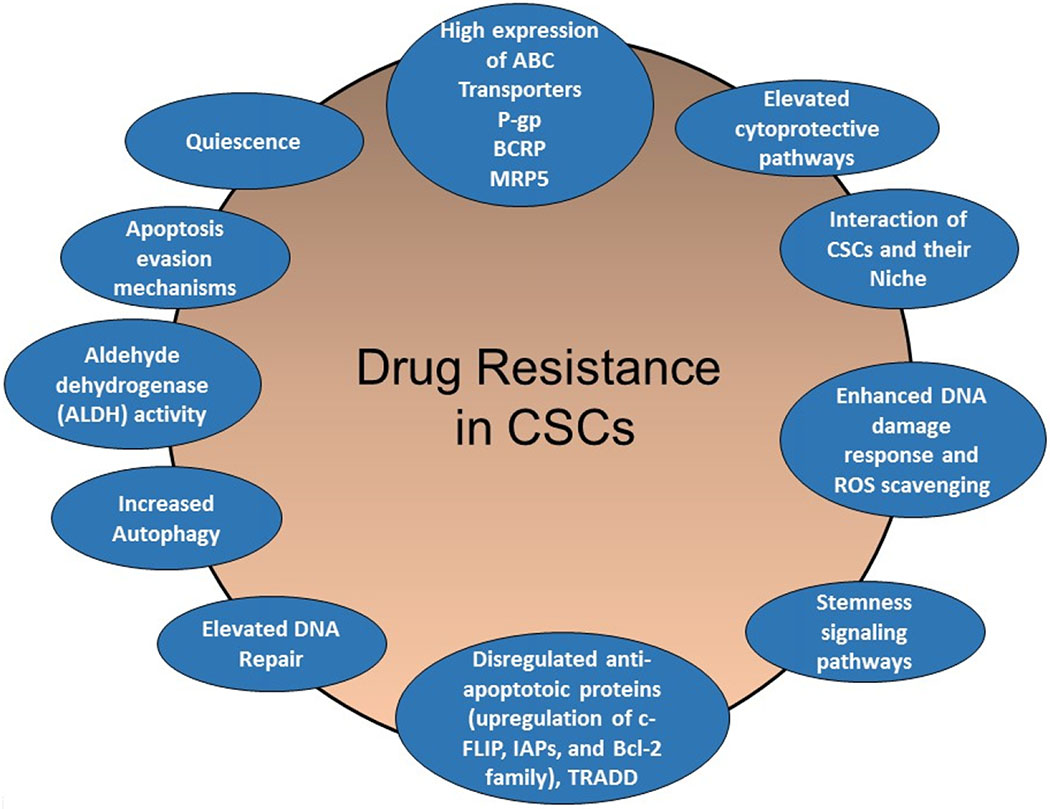
Schematic presentation of CSC-mediated therapy resistance to cancer. Activation of cell survival pathways, quiescence, increased drug efflux, impairment of the apoptotic pathway, increased DNA damage repair, increased detoxifying activity, and increased scavenging of free radicals are possible contributors to the therapy resistance of CSCs

**Figure 4.**
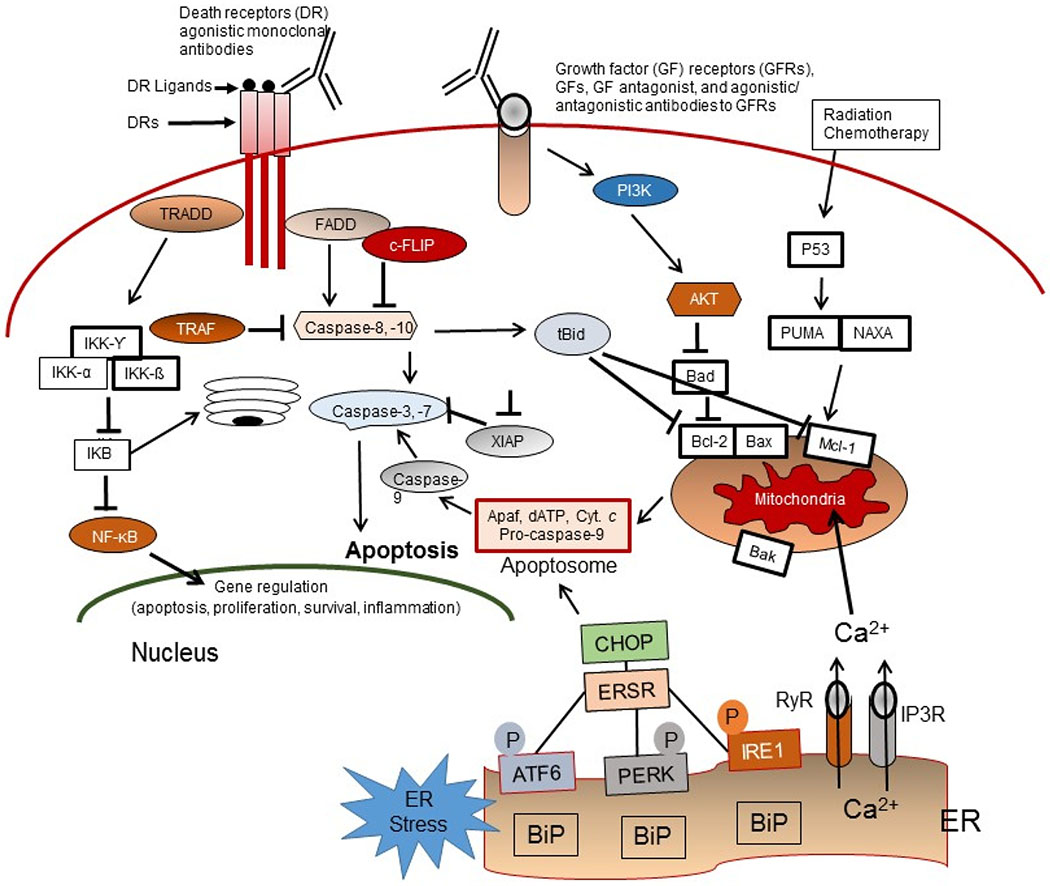
Apoptosis signaling pathways. Overview of the intrinsic (mitochondrial), extrinsic or death receptor (DR), and ER-stress (ERS)-mediated apoptosis pathways in response to the molecular action of anticancer agents as well as the TRADD/NF-κB survival pathway, the growth factor (GF) receptors, and PI3K/Akt pro-survival signaling axis in CSCs

In this review article, I discuss the contribution of particular drug resistance mechanisms and signaling pathways that control CSC maintenance. Understanding these mechanisms is important for overcoming drug resistance in these cells [13–15,23,34]. The major mechanisms of chemotherapeutic and apoptotic resistance in CSCs are summarized in Figures 3 and 4.

## Cancer stem cells signaling pathways

It is well documented that various cancers are initiated from CSCs and these cells are responsible for patient resistance to therapies [11,43–46]. Furthermore, due to the heterogeneity, high diversity, and plasticity of CSCs, developing useful therapeutics to target them has been difficult. Evidence also suggests the possibility of non-CSC reprogramming and dedifferentiation to CSCs (Figure 1), resulting in a further increase in the complexity and diversity of resistance mechanisms in tumors. Therefore, because of this complexity, potent and effective anticancer treatment must eradicate both CSCs and the entire bulk of the heterogeneous tumor population, as well as avoid triggering tumor cell dedifferentiation of non-CSCs to CSCs or cancer stem-like cells. Various signaling pathways including the Notch, Hedgehog, Wnt/β-catenin, PI3K/Akt/ mTOR (mTORC1 and mTORC2), maternal embryonic leucine zipper kinase (MELK), TGF-β, STAT, and Hippo-YAP/TAZ among others are activated and operational in CSCs [11,46–49]. These pathways and the cancer stem cell markers including CD133, CD44, Oct4, Sox2, Nanog, and ALDH1A1 maintain CSC properties [11,34,50,51].

Another important factor, epigenetic modification of CSCs, could result in phenotypic and functional heterogeneity among the cell populations within various solid tumors. Accumulating evidence indicates that the enhancer of zeste homolog 2 (EZH2), which is the catalytic subunit of Polycomb repressive complex 2 (PRC2) and has histone methyltransferase activity, is upregulated in CSCs and has an important function in their expansion and maintenance [52]. Furthermore, histone deacetylases (HDACs) 1, 6, 7 and 8, known to deacetylate transcription factors and other cellular proteins, are overexpressed in CSCs [53,54].

Significantly, it has been shown that that hypoxia-driven CSC enrichment results from a dedifferentiation process in breast cancer and that hypoxia-inducible factors (HIFs) are required for chemotherapy resistance in breast CSCs (BCSCs) [55], glioblastoma CSCs [56] and other solid tumors [57]. Interestingly, the dedifferentiated CSCs display multidrug resistance (MDR) via PERK-Nrf2 signaling pathway [58]. Lee et al. [56] have demonstrated that temozolomide (TMZ)-triggered HIF1α/HIF2α upregulation plays a critical role in converting non-stem glioma cells to acquire stem-like characteristics, and that knockdown of HIF1α/HIF2α suppressed the interconversion between non-stem glioma cells and GSCs post-therapy [56].

Another crucial signaling protein, MELK, a serine/threonine kinase, is upregulated in human cancers and CSCs [59] and plays significant roles in the survival and other known characteristics of CSCs including drug resistance and tumor recurrence. Kim et al. [60] demonstrated that MELK phosphorylates the FOXM1 transcription factor and that EZH2 is targeted by the MELK/FOXM1 complex, which promotes CSC resistance to radiation, and that OTS167 (an inhibitor of MELK) effectively eliminates CSCs from small cell lung cancer (SCLC) [61].

The CSC characteristics that may help in the development of anti-CSC therapies include specific cell surface markers and particular networks of transcription factor signaling, aberrant signaling pathways, epigenetic alterations, reprograming and plasticity, interaction with the microenvironment and CSC niche, and using particular metabolic pathways [25,43,60–64].

## Apoptosis pathways and resistance to apoptosis in CSCs

Apoptosis resistance and overexpression of anti-apoptotic proteins are necessary for CSC survival and self-renewal capacity. To discuss the mechanisms of apoptosis and cancer-related chemotherapeutic drug resistance, apoptosis signaling pathways are first described. Cancer cells and CSCs avoid apoptosis, but apoptosis in these cells occurs through several signaling pathways in response to chemotherapeutic agents and various apoptotic stimuli. Much evidence shows that mutations in normal stem cells (NSCs) alter these cells into CSCs (Figure 1), enabling them to avoid apoptosis and leading to tumor formation.

Three major apoptosis pathways are the extrinsic or cell surface death receptors pathway, the intrinsic or mitochondrion-initiated pathway, and endoplasmic reticulum (ER) stress-mediated pathway control of apoptosis (Figure 3) [65–74]. The death receptor mediated or extrinsic apoptotic pathway is initiated by the binding of death receptors (DRs) with their ligands [i.e., interaction of Fas/Fas ligand, tumor necrosis factor-α (TNF-α)/TNF receptor 1 (TNFR1), TRAIL/DR4, or TRAIL/DR5] (Figure 2). Ligand and DR interaction triggers recruitment of FADD and initiator pro-caspase-8 or -10 to form the death-inducing complex (DISC), resulting in activation of these pro-caspases to caspases-8 and -10 by an autocatalytic process. These caspases in turn activate the effector caspases-3, -6, and -7 and cause degradation of the downstream proteins leading to apoptosis. Caspases-8 and -10 cleave the pro-apoptotic Bcl-2 family member Bid to truncated tBid linking the extrinsic apoptosis pathway to the intrinsic or mitochondrial pathway and trigger cytochrome *c* release from mitochondria [63–67]. The DR initiated apoptosis pathway is suppressed by the anti-apoptotic protein c-FLIP, which inhibits DISC formation and activation of caspases-8 and -10 and blocks apoptotic.

In the mitochondrial or intrinsic apoptosis pathway, various apoptotic stimuli (e.g., DNA damaging agents, radiation, conventional chemotherapeutic drugs, and small molecule anticancer compounds) trigger mitochondrial outer membrane permeabilization (MOMP). This process is initiated by activation of Bcl-2 homologous pro-apoptotic proteins (e.g., Bax, Bak, and Bad) and the Bcl-2 homology domain-3 (BH3)-only family of proteins including Bid, Bim, or PUMA [65–71], and provides an interactive protein network with mitochondria, which leads to the release of apoptosis triggering factors. The apoptosis inducing factors include certain caspases, Smac/DIABLO, and other factors from the mitochondrial intramembrane space to the cytosol. After release from mitochondria, cytochrome *c* and dATP bind to apoptotic proteinase-activating factor-1 (Apaf-1) to form the apoptosome, and this complex triggers procaspase-9 autoactivation. The active caspase-9 in turn activates caspases-2, -3, -6, -7, -8, and -10, leading to degradation of cellular proteins and resulting in apoptosis [6–71].

The third main apoptosis pathway is the endoplasmic reticulum (ER)-mediated apoptosis pathway. The ER promotes the correct folding of proteins and mediates ER-associated degradation of unfolded or misfolded protein. Dysregulation of ER functions triggers an accumulation of unfolded or misfolded proteins in the ER lumen, leading to ER stress (ERS), which induces the unfolded protein response (UPR) or the ERS response (ERSR), resulting in restored homeostasis or apoptosis [75,76].

CSCs display resistance to apoptosis by upregulating the expression of anti-apoptotic proteins including the cellular FLICE-inhibitory protein (c-FLIP), the Bcl-2 family of proteins, and inhibitor of apoptosis proteins (IAPs) [11,77,78]. CSCs upregulate c-FLIP expression and are resistant to TNF-related apoptosis-inducing ligand (TRAIL)-induced apoptosis [79]. Overexpression of IAPs also plays a crucial role in resistance to TRAIL and chemotherapeutic agents, as well as CSC apoptosis [80].

## Mechanisms of CSCs death resistance

Several mechanisms trigger drug resistance and make CSCs refractory to apoptosis. Characterizing the mechanisms that prevent apoptosis and identifying useful therapeutic targets to increase apoptosis in CSCs are particularly significant for successful cancer therapy. These mechanisms are discussed in detail in the following sections.

## Multidrug resistance transporters in CSCs

Several ABC transporters including P-glycoprotein (P-gp, MDR1, ABCB1), multidrug resistance protein 1 (MRP1, ABCC1), breast cancer resistance protein (BCRP, ABCG2) [81–84], and MRP5/ABCC5 [83], have been extensively investigated as multidrug resistance transporters in tumors. Overexpression of these proteins in several solid tumor types, acute myeloid leukemia (AML), and myeloma lead to ATP-dependent efflux of a wide range of conventional chemotherapeutic agents as well as many molecular-targeted cancer drugs, resulting in lower drug levels in the resistant cells below the amount required to trigger cell death [81–84].

Conclusive evidence shows that CSCs in various solid tumors and hematological malignancies upregulate these ABC transporters, resulting in drug resistance in these cells [85]. Wang et al. [86] reported that Panc-1 pancreatic CSCs displayed resistance to gemcitabine, upregulated expression of CD133/CD44/Oct4/Nestin compared to the parental Panc-1 cells, and overexpressed P-gp and anti-apoptotic proteins. In glioblastoma CSCs, epigallocatechin gallate (EGCG) treatment downregulated P-gp overexpression but not that of ABCG2 or O6-methylguanine-DNA methyltransferase (MGMT) and increased the cytotoxic effect of TMZ [55]. Wilson et al [84] isolated melanoma cancer stem cells (MCSCs) and demonstrated that ABCB5 maintains these cells.

## PI3K/Akt/mTOR signaling pathway plays a crucial role in CSCs

This pathway functions in many important cellular processes and contributes to drug resistance in cancer. Several studies have demonstrated that upregulation of PI3K/Akt/mTOR plays a pivotal role in the CSC maintenance [87–91]. Emerging data suggest that the PI3K/Akt/mTOR signaling pathway could be a rational and promising target for the development of anti-CSC drugs including salinomycin, metformin, silibinin E1201, rottlerin, and torin [89]. Moreover, the antidiabetic drug metformin, an inhibitor of PI3K/Akt/mTOR signaling, effectively reduced temozolomide (TMZ) resistance in CSCs [92]. Furthermore, the combination of metformin with sorafenib as a RAF inhibitor also significantly reduced CSC oxidative stress, efflux pump activity, and synergistically killed these cells [93]. CSCs heavily rely on mitochondrial oxidative phosphorylation. Metformin has been shown to use this metabolic weakness and enhance CSC sensitivity to conventional cancer chemotherapies, overcome drug resistance and increase treatment efficacy [94].

## Dysregulated anti-apoptotic proteins in CSCs in the Bcl-2 family of proteins

Apoptosis evasion and the capacity of cancer cells to self-replicate, proliferate, and metastasize are distinct hallmarks of cancer [11,95]. In various cancers, several steps within the extrinsic and intrinsic apoptotic pathways in CSCs may be dysregulated [11,96]. However, the abnormal expression levels as well as levels and ratios of apoptotic and anti-apoptotic proteins and their contribution to drug resistance in CSCs have not been well characterized and delineated in detail. Bcl-2 family proteins consist of the anti-apoptotic molecules Bcl-2, Bcl-XL and Mcl-1 and the pro-apoptotic proteins Bax, Bak, Bid, Bim, Bik, Noxa and Puma [97,98]. Increased levels of Bcl-2 family proteins were shown in CSCs and high levels of these proteins are associated with the resistance of CSCs to apoptosis and anticancer drugs [99,100]. This resistance is partly due to the ratio of anti- to pro-apoptotic protein levels, which increases cell survival [99]. Moreover, aberrantly increased expression of nuclear factor erythroid 2-related factor 2 (Nrf2), the redux-sensing transcription factor promotes CSC survival by elevating transcription of ABCG2, Bcl-2, and Bmi-1, a member of the Polycomb Repressor Complex (PRC1) genes [101]. Since expression of the Bcl-2 family of anti-apoptotic proteins is significant for cell survival and resistance to apoptosis and drugs in CSCs, therapeutic interventions to eliminate CSCs using inhibitors of these proteins have been actively pursued.

## TRADD and NF-κB

The tumor necrosis factor receptor 1 (TNFR1)-associated death domain protein (TRADD) is an adaptor protein in TNFR1 signaling and participates in NF-κB activation as well as survival signaling in CSCs [65] downstream of DR4, DR5 (Figure 2). Moreover, TRAIL has been shown to promote the formation of the intracellular Complex II composed of FADD, TRADD, caspase-8, caspase-10, RIP1, TRAF2 and IKK-γ [102]. Cancer cells and CSCs often have constitutively activated transcription factor NF-κB that promotes expression levels of apoptosis inhibitory proteins and drug resistantance proteins and provides enhanced survival and resistance to therapies in cancer cells. Upregulated expression of TRADD is sufficient to activate NF-κB in glioblastoma (GBM) cancer stem cells (GSCs) [103]. Furthermore, cytoplasmic TRADD expression has been shown to be significantly associated with worse progression-free survival (PFS) in GBM patients. Knockdown of TRADD by shRNA in GSCs triggered decreased NF-κB activity and reduced the viability of these cells, revealing that TRADD is required for maintenance of GBM stem cell populations [103]. NF-κB signaling plays an important role in the maintenance of CSCs [104]. In advanced ovarian cancers, NF-κB signaling via the RelB transcription factor supports directly regulating the cancer stem-like associated enzyme aldehyde dehydrogenase (ALDH) [105]. Additionally, the NF-κB signaling pathway plays a critical role in the chemoresistance of gastric cancer stem cells [105]. NF-κB activity supports CSC maintenance and reduced sensitivity to NF-κB inhibitors, indicating that high activity of NF-κB plays a critical role in the survival of CSCs [106].

## Role of the IAP family in CSC drug and apoptosis resistance

The IAP family consists of several proteins including survivin, IAP1, cIAP2, X-linked inhibitors of apoptosis (XIAP), ML-IAP, NAIP, and ILP-2 [70–72]. IAPs inhibit the activity of caspases-3, -7, and -9 and participate in the evasion of cancer cells from apoptosis [77,80]. Overexpression of IAP family proteins has been shown in solid tumors and hematological malignancies and causes resistance to apoptosis, chemotherapeutics agents, and radiation therapy as well as causing poor prognoses [77]. IAPs function through interactions of their BIR (baculoviral IAP repeat) protein domains and these interactions are antagonized by Smac/Diablo, an inverse regulator for the inhibitors of IAPs and induction of apoptosis [77,80]. Survivin plays a role in CD133+ cell chemoresistance to 5-fluorouracil (5-FU) in colon CSCs and a survivin inhibitor may be a new targeted agent to effectively treat CD133+ colon cancer [107].

The critical roles of IAPs in maintaining CSCs and the importance of IAP inhibitors being more effective for CD133+ stem-like medulloblastoma (MB) CSCs has been shown [108]. Holt et al. [109] showed that pharmacological downregulation of XIAP in pediatric tumor cells triggers apoptosis and sensitizes cells to cytotoxic agents. Furthermore, Ji et al. [110] have shown that XIAP, a member of the IAP family, has a critical role in maintaining CSCs in nasopharyngeal carcinoma (NPC) stem cells. These authors showed that XIAP regulates the stability of the CSC marker Sox2, which is important for the maintenance and self-renewal of NPC CSCs. The important role of IAPs in CSCs was also demonstrated by Janzen et al. [111] showing that the cIAP inhibitor Birinapant overcomes platinum resistance in a CSC population of ovarian cancer in vivo, indicating that IAPs may play a significant role in cancer drug resistance and recurrence.

## c-FLIP overexpression in CSCs

c-FLIP is a catalytically inactive caspase-8/-10 homolog and a master anti-apoptotic protein that suppresses cytokine- and chemotherapy-induced apoptosis and causes resistance to these agents [112]. c-FLIP is expressed as long (c-FLIPL), short (c-FLIPS), and c-FLIPR splice variants in human cells. c-FLIP binds to FADD and/or caspases-8 or -10 and TRAIL receptor 5 (DR5) and prevents Death-Inducing Signaling Complex (DISC) formation. Moreover, c-FLIPL and c-FLIPS are also known to have multifunctional roles in various signaling pathways, as well as activating and/or upregulating several cytoprotective and pro-survival signaling proteins including Akt, ERK, and NF-κB. It is known that Ku70, a protein that repairs DNA breaks, stabilizes c-FLIP, which is regulated by acetylation [113]. Several reports have demonstrated that c-FLIP isoforms maintain the survival and resistance of CSCs to apoptosis and anti-cancer therapies [79,114,115]. CD133, a CSC marker that plays a role in CSC tumorigenesis, metastasis and chemoresistance, can also upregulate the expression of c-FLIP in CD133+ cells, thus inhibiting apoptosis [116,117].

## Aldehyde dehydrogenase (ALDH) activity

Aldehyde dehydrogenase (ALDH) isoforms consist of a superfamily of enzymes that detoxify a variety of endogenous and exogenous aldehydes and high ALDH activity has been frequently used as a selectable marker for CSCs [118,119]. Much evidence suggests that ALDH may be used as a marker for CSC self-renewal, proliferation, differentiation, and resistance to drugs [118–120]. It is well documented that the ALDH protein family is a hallmark of CSCs and ALDH1A1 is the most studied ALDH isoform [121,122]. Expression of ALDH1 protein in CSCs is a negative prognostic indicator and predictor of poor clinical outcome in cancer patients, and high ALDH activity has been attributed to chemoresistant CSCs in different tumor types [118,119,121]. Overall, substantial data indicate a critical role of ALDH, particularly ALDH1, in CSC biology and therapy resistance [118–122]. Therefore, inhibition of ALDH activity may be a rational and potentially useful therapeutic strategy for targeting CSCs with the aim of increasing the efficacy of cancer therapies.

## Enhanced DNA damage response and ROS scavenging in CSCs

Much evidence has shown that CSCs are resistant to DNA damaging therapies by regulation of the cell cycle, increasing DNA repair capacity, and effective scavenging of reactive oxygen species (ROS) [123–126]. DNA-damage response (DDR) is considered a significant source of resistance to DNA-damaging treatments and CSCs, and check point inhibitors to sensitize CSCs to DNA-damaging treatments have been developed [127]. Interestingly, DDR appears as a relevant target to sensitize cancer cells and cancer stem cells to classical radio- and chemotherapies as well as to overcome resistance [127]. Bartucci et al. [128] reported that in NSCLC, chemotherapy targeting the DNA damage checkpoint (CHK1) signaling in CSCs was p53 independent and caused cell cycle arrest, more efficient DNA damage repair, and enhanced cell survival compared to the bulk of the tumor cell population. Moreover, targeting CHK1 and PARP1 may provide an effective anti-CSC strategy [126].

## Autophagy as a cytoprotective and drug resistance mechanism in CSCs

Autophagy is a catabolic pathway which is characterized by autophagosome formation and triggers tumor cell survival and drug resistance [129–131]. Autophagy is critical as a survival mechanism in tumors having defects in the apoptotic signaling pathways, and CSCs show a high level of autophagy, which contributes to their survival and therapy resistance [131–133]. Autophagy also determines cell fate by targeting degradation of key transcription factors, including p53 and FoxO3A, or by enforcing quiescent growth arrest [131]. Apart from promoting resistance to chemotherapy, high levels of autophagy in CSCs maintains their pluripotency, allows them to cope with low nutrients and hypoxia in the tumor microenvironment, regulates CSCs migration and invasion, and helps them escape immunosurveillance [132]. Beclin 1, a Bcl-2 homology 3 (BH3) domain only protein, is an essential initiator of autophagy and a critical determinant of whether cells undergo autophagy or apoptosis [134]. The BH3 domain of Beclin 1 interacts with Bcl-2 family members. Therefore, the role of Bcl-2 in inhibiting apoptosis and autophagic cell death makes the Bcl-2 protein and autophagy manipulation excellent targets and strategies to inhibit drug, anti-apoptotic, and autophagy-related resistance mechanisms.

## Conclusion

It well documented that CSCs achieve the hallmarks of malignancy including self-renewal and replicative immortality; resistance to chemotherapeutic agents, radiotherapy, and cell death; invasiveness and tumor recurrence. CSC-related drug resistance mechanisms might be important for predicting patient response to therapies and guiding treatment selection for various tumors. A challenging task for the development of CSC-specific therapeutics is identifying specific biomarkers of CSCs to analyze their population during the course of tumor treatment. Taken together, the foregoing discussion reveals that CSCs can contribute to tumor resistance to chemotherapeutic agents and cell death, provide a better understanding of the molecular mechanisms underlying CSC unresponsiveness to therapies, and may lead to the identification of specific therapeutics and novel strategies to increase the sensitivity of CSCs to cancer therapeutics.

## Abbreviations:

Apaf-1: Apoptotic Proteinase-Activating Factor-1
AIC: Apoptosis Inhibitory Complex
BH3: Bcl-2 Homology Domain-3
CSCs: Cancer Stem Cells
c-FLIP: cellular FLICE (FADD-like IL-1β-converting enzyme)-Inhibitory Protein
DISC: Death-Inducing Signaling Complex
DNA-PK: DNA-Dependent Protein Kinase
FADD: Fas-Associated Death Domain
ER: Endoplasmic Reticulum
DRs: Death Receptors
GBM: Glioblastoma Multiforme
mTOR: Mammalian Target of Rapamycin
MELK: Maternal Embryonic Leucine Zipper Kinase
Nrf2: Nuclear Factor Erythroid 2-Related Factor 2
P-gp: P-glycoprotein
PDA: Pancreatic Ductal Adenocarcinoma
PTP: Permeability Transition Pore
PERK: Protein Kinase R (PKR)-like Endoplasmic Reticulum Kinase
TNFR1: TNF Receptor 1
TNF-α: Tumor Necrosis Factor-α
TRAIL: TNF-Related Apoptosis-Inducing Ligand
TRADD: TNFR1-Associated Death Domain Protein (TRADD)
UPR: Unfolded Protein Response
Hh: hedgehog
